# Contamination of Tea and Tea Infusion with Polycyclic Aromatic Hydrocarbons

**DOI:** 10.3390/ijerph15010045

**Published:** 2017-12-28

**Authors:** Alicja Zachara, Dorota Gałkowska, Lesław Juszczak

**Affiliations:** 1 Department of Food Analysis and Evaluation of Food Quality, Faculty of Food Technology, University of Agriculture in Krakow, Balicka 122, 30-149 Krakow, Poland; azachara@poczta.onet.pl (A.Z.); d.galkowska@ur.krakow.pl (D.G.); 2 Laboratory of Food Hygiene and Nutrition, Voivodeship Sanitary-Epidemiological Station in Rzeszow, Wierzbowa 16, 35-959 Rzeszow, Poland

**Keywords:** tea, tea infusion, PAHs, HPLC-FLD, QuEChERS

## Abstract

The aim of this work was to validate the method of determination of polycyclic aromatic hydrocarbons (PAHs), i.e., benzo(a)pyrene and sum of benzo(a)pyrene, benz(a)anthracene, benzo(b)fluoranthene and chrysene in different types of tea, as well as to assess the transfer of these contaminants from tea to tea infusion. The research materials were popular types of black, green, red and white tea. Quantitative and qualitative determination of PAHs was performed by High Performance Liquid Chromatography with fluorimetric detection (HPLC-FLD). The samples were prepared by QuEChERS (Quick, Easy, Cheap, Effective, Rugged and Safe) technique followed by cleaning-up by dispersion solid-phase extraction (d-SPE). Values of limit of detection and limit of quantification obtained in the validation of the method were lower than the respective maximum values given in Commission Regulation (EU) No. 836/2011. The level of contamination of popular teas commercially available on the Polish market with PAHs is similar to that of teas available in other countries, with a very large variation in the concentration of each of the compounds. The highest benzo(a)pyrene and Σ4PAHs contents (209 ± 42 μg/kg and 756 ± 151 μg/kg, respectively) were found for black tea leaves. The transfer of Σ4PAHs from black tea to tea infusions was 0.48%, while it was 1.55–1.72% for red, white and green teas.

## 1. Introduction

The tea leaf infusion (*Camellia sinensis*) is one of the most widely consumed beverages in the world due to its palatability, the tradition of preparing and drinking, and the wide variety of commercially available tea species. In addition to the sensory characteristics such as color, taste and aroma, the popularity of tea infusion is determined by its health-promoting effects, which depend on the amount and bioavailability of bioactive compounds in the dried tea [1]. The classification of tea is based on many criteria, including: country of origin, region of cultivation, part of the tea tree, the form of finished product or the tea processing technology, where the latter is the most famous criterion. There are black, green, red, and white teas; however, for white tea there is no general definition. The chemical composition of the finished tea product depends on both chemical composition of the raw material (plant) and on the tea processing. The former is determined by environmental factors including cultivation method, atmospheric conditions and the harvesting period [2]. Moreover, environmental pollution can contribute to contamination of tea with heavy metals, dioxins, pesticide residues and polycyclic aromatic hydrocarbons (PAHs) [3,4]. The latter can be adsorbed with dust particles on tea leaves and buds or can be respired by plant from air [5,6]. Roasting or smoking of tea leaves can also be a source of PAHs in a finished product [7,8,9,10].

According to the current state of knowledge, polycyclic aromatic hydrocarbons have different toxicities: they can cause dangerous in vivo effects, including cytotoxic, immunotoxic, genotoxic, teratogenic and cancerogenic ones [11,12,13,14]. Benzo(a)pyrene is a well-known substance classified by the International Agency for Research on Cancer (IARC) into group 1 of carcinogens—i.e., factors with proven harmful (carcinogenic) effects on the human body—while benzo(b)fluoranthene, chrysene and benz(a)anthracene are classified into group 2B—i.e., group of compounds with a possible carcinogenic effect on the human body [13]. Polycyclic aromatic hydrocarbons are easily absorbed in the human gastrointestinal tract, especially when there is a large amount of fats in the diet. These compounds do not accumulate in the human body; they show relatively low acute toxicity, but very high chronic toxicity [11]. In the first stage of metabolism, PAHs are oxidized by cytochrome P450 oxidase to highly reactive hydroxyl and epoxy PAH derivatives (including diol-epoxides). The oxidized intermediates, in particular epoxy derivatives, can form covalent bonds with DNA, causing its damage and eventual carcinogenesis. In the second stage of metabolism, hydroxy PAH derivatives are coupled with glucuronic or sulfuric acid in the presence of specific transferases. In this form, they are excreted from the body, primarily with bile and to a small extent with urine [11,13,15].

The benzo(a)pyrene (BaP), benz(a)anthracene (BaA), benzo(b)fluoranthene (BbFA) and chrysene (Chr) are limited in foods such as oil, smoked meat and fish products, processed cereal-based foods and baby foods, dietary supplements, cocoa beans, dried herbs and spices [16,17]. European Food Safety Authority (EFSA) has not introduced legislation on maximum permissible PAHs content in tea and fruit teas, although setting such limits has been debated. It was considered that there has not been enough research on the transferring of PAHs from tea to tea infusion in order to estimate the risk of consumer exposure. However, further research is important to estimate the level of PAH contamination of commonly consumed teas [18,19].

According to available scientific studies determination of PAHs in tea is difficult due to the high content of interfering substances such as caffeine, polyphenols, sugars, organic acids and pigments, including chlorophylls. Hence, modern techniques of extraction and purification of samples are used [4,10,20,21,22,23]. One of the modern techniques for purifying samples for pesticide as well as polycyclic aromatic hydrocarbons analyses is QuEChERS (Quick, Easy, Cheap, Effective, Rugged and Safe). The main advantages of this technique are the speed and ease of sample preparation and environmental safety due to the low consumption of chemical reagents, as well as lower cost of analysis compared to other methods [4,21,24].

The aim of this work was to validate the method of determination of polycyclic aromatic hydrocarbons (PAHs), i.e., benzo(a)pyrene (BaP), benz(a)anthracene (BaA), benzo(b)fluoranthene (BbFA) and chrysene (Chr) (individually and as a sum of the four PAHs (Σ4PAHs)), as well as to assess the transfer of these contaminants from tea to tea infusion.

## 2. Materials and Methods

### 2.1. Samples

The sampling plan was made according to the classification of tea most often used by consumers, i.e., type of tea: black, green, red and white. Teas branded in this way possess characteristic sensory and health attributes and this classification is related to the method of tea processing. Samples of black, green, red and white leaf teas (28 samples) were of different manufacturers or distributors and were purchased from low and medium price range in local shops. According to the information on the packaging the products consisted mostly of mixtures of teas. The primary three samples from the bath of each kind of tea (10 black tea samples, 6 each green, red and white tea samples) were collected into bulk sample. Two laboratory samples for each type of determination were weighed out of the bulk sample. They were analysed both in a dry form and in form of tea infusions. Before analysis the samples were stored according to the recommendations given on packages, i.e., in a dry place away from light.

### 2.2. Reagents

For the analysis the following reagents were used: acetonitrile (of HPLC purity) (Merck, Darmstadt, Germany), QuEChERS Bulk Sodium Chloride, SampliQ Anhydrous Magnesium Sulfate for QuEChERs and Primary Secondary Amine (PSA) SPE Bulk Sorbent (all from Agilent Technologies, Santa Clara, CA, USA). Water of HPLC purity was from LiChrosolv (Merck, Darmstadt, Germany). The certified standard PAH Solution Mix from AccuStandard (New Haven, CT, USA) consisted of PAH solution in methanol-dichloromethane (MeOH-DCM), with 200.6 μg/mL of BaP, 197.8 μg/mL of BaA, 198.8 μg/mL of BbFA and 199.0 μg/mL of Chr.

### 2.3. Apparatus

High Performance Liquid Chromatography with fluorimetric detection (HPLC-FLD) was used for the determination of each of the PAHs. The Nexera X2 (Shimadzu Corporation, Kyoto, Japan) chromatographic system was used, which consisted of SIL-30AC auto-sampler, two LC-30AD pumps, Prominence RF-20Axs fluorescence detector, connected to Lab Solution software ver. 5.57 (Shimadzu Corporation, Kyoto, Japan). The Hypersil Green PAH (Thermo Scientific, Waltham, MA, USA) column (250 × 4.6 mm, I.D., 5 μm) and guard column (10 × 4.0 mm, I.D., 5 μm) were used.

### 2.4. Methods

#### 2.4.1. Sample Preparation

**Figure 1:**
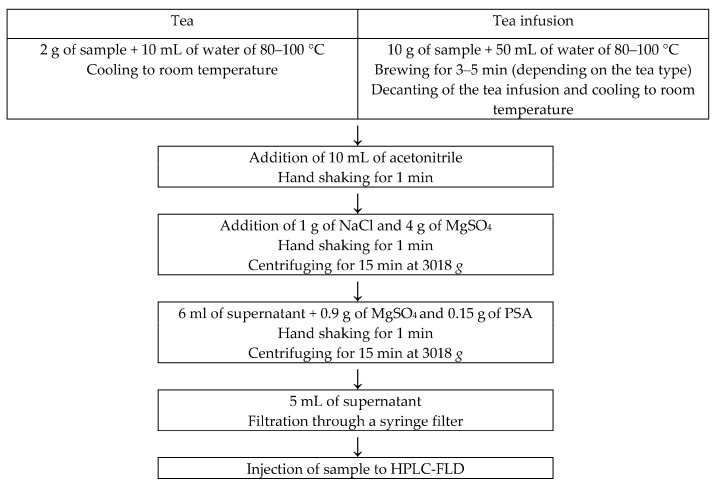
Procedure diagram for sample (tea and tea infusion) preparation for the determination of polycyclic aromatic hydrocarbons (PAHs) using High Performance Liquid Chromatography with fluorimetric detection (HPLC-FLD).

Three packages of tea of the same batch were taken and their content was mixed, without grinding. The procedure diagram for sample (tea and tea infusion) preparation for the determination of PAHs using HPLC-FLD is presented in Figure 1.

#### 2.4.2. Preparing the Tea Infusion

The amounts of tea and water used to brew the tea have been established experimentally, while the temperature of the brewing water and the brewing time were adjusted according to the instructions on the product packages. The dried tea was infused in freshly boiled water of appropriate temperature and brewed without cover. The water temperature and time of brewing was as follows: 100 °C/5 min, 80 °C/3 min, 95 °C/4 min and 85 °C/3 min for black, green, red and white tea, respectively. On the basis of preliminary analyzes of dried black and white tea, i.e., samples with the highest and lowest PAH content, respectively, it was found that the PAH content in the infusions made of dried tea and water used in a 2:250 mass–volume ratio (commonly used by consumers) is very low and is below LOD. Therefore, further analyses were carried out with the increasing ratio of dried tea to water, so that the PAH content determined in the infusion was above the LOD. As a result of the experiment, it was found that the amount of PAHs determined in both the black and the white tea infusions prepared by infusion of 10 g of dried tea in 50 mL of water was higher than LOD (Table 1). Therefore, the 10:50 tea (g) to water (mL) ratio was used in the study. After brewing, the tea infusion was decanted (without filtration) and allowed to cool down to room temperature. The results of analysis of the blank samples carried out for each series of samples showed no contamination of the water used for brewing.

#### 2.4.3. Extraction and Purification of the Hydrocarbon Fraction

In the initial stage of the research, a comparison of two methods of extraction and purification of the PAHs was made [25]: the method involved use of a glass column filled with aluminum oxide [26] and the QuEChERS procedure. For this purpose, a sample of black tea was used. The results of the determinations, corrected for recovery, are expressed as the content of individual PAHs in the sample under study.

Procedure of sample (tea and tea infusion) preparation by QuEChERS technique for the determination of PAHs is presented in Figure 1. An aliquot of 10 mL of acetonitrile was added to the sample and the sample was hand shaken for PAHs extraction. Then sodium chloride and anhydrous magnesium sulphate were added and then the sample was hand shaken and centrifuged (MPW–350R, MPW Med. Instruments, Warsaw, Poland) in order to separate the phases into the aqueous and the organic ones. The organic extract was purified by adding anhydrous magnesium sulphate and PSA amine sorbent followed by hand shaking and centrifuging. The resulting supernatant was filtered through a syringe filter (0.2 μm, Alchem Grupa Sp. z o.o., Toruń, Poland) [21,27,28].

#### 2.4.4. HPLC-FLD Analysis

The sample was injected into HPLC using an auto-sampler. The temperature of the column was maintained constant at 20 °C. The mobile phase was constituted of acetonitrile and water. The elution conditions applied were: 0–16 min, 60% of acetonitrile; 16–45 min, 100% of acetonitrile; 45–49 min, 60% of acetonitrile. The flow rate was 1.0 mL/min. The effluents were monitored using the following excitation and emission (Ex/Em) wavelengths: 260/420 nm for BaA and Chr and 290/430 nm for BaP and BbFA.

##### Preparation of Calibration Curves

Calibration curves were performed by external standard method, using the standard substance in five concentrations in the range of 0.10–10.00 ng/mL. The curves were plotted as linear functions: y = a∙x, where x is the peak area of the standard substance and y is of the measured signal [26].

##### Determination of the Working Range and Linearity of the Method

For each of the PAH calibration curves the coefficients of variation for concentration limits were calculated and then the F-Snedecor test was used for testing homogeneity of coefficients of variation at a 0.05 significance level. Correlation coefficient (r) was also calculated. The limit of detection (LOD) and the limit of quantification (LOQ) were calculated according to the guidelines given in the European Reference Laboratory (EURL) experts’ report concerning the PAHs [29]. For this purpose ten determinations of PAH content in a dried tea sample of the lowest PAH content were made. The LOD and LOQ were calculated according to the following formulas: LOD = 3.9 × S_y,b_/b and 3.3 × LOD respectively, where S_y,b_—standard deviation of the blank signals, b—slope of the calibration curve. The sensitivity of the method was measured as the slope of the calibration curve.

##### Measurement of Samples

The PAH determination was performed by a validated method meeting the criteria of Commission Regulation (EU) No. 836/2011 [30]. The measurement procedure consisted of dosing the following samples: blind samples, samples, two calibration solutions (1.00 and 9.00 ng/mL) and samples enriched with certified standard sample.

### 2.5. Statistical Analysis

Results obtained in the procedure of validation of the method were tested by Dixon Q test for identification and rejection of outliers. Then, for each of PAHs concentrations the following statistical parameters were calculated: recovery factor (correctness), variance, standard deviation, coefficient of variation, standard and expanded uncertainties, confidence interval and relative standard deviation of repeatability. The evaluation of the significance of differences between mean values of PAH concentration was made using Tukey multiple comparison test at a 0.05 significance level. All statistical procedures were computed using Statistica version 12.0 (StatSoft, Krakow, Poland).

## 3. Results and Discussion

### 3.1. PAH Content of Tea

The values of the limit of detection (LOD) and the limit of quantification (LOQ) complied with the criteria set out in the Commission Regulation (EU) 836/2011 [30] (Table 2). In the concentration range of 0.10–10.00 ng/mL the calibration curves for each of the standard substances were linear, with values of correlation coefficient higher than 0.998. In order to assess the repeatability of the method, twenty analyses of BaP, BaA, BbFA and Chr contents in black tea were performed. The criteria of Commission Regulation (EU) 836/2011 [30] were fulfilled, since the values of RSDr were lower than 20% and the Horrat coefficient was lower than 2 (Table 2). 

The correctness (accuracy) of the method was determined by performing a full analytical procedure in six replicates for black tea samples enriched with a mixture of BaP, BaA, BbFA and Chr at three concentration levels: 5.0 μg/kg and 40.0 μg/kg and 250 μg/kg. The recovery mean values were in the range of 50.75–93.30% (Table 2). Expanded uncertainty was also calculated by multiplying the standard uncertainty with a coverage factor k = 2 and its value for all of the PAHs determined was 20%.

**Figure 2:**
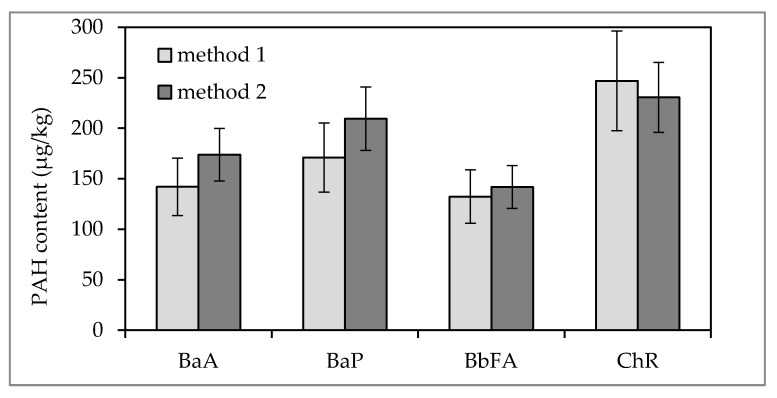
PAH contents in tea as determined by HPLC-FLD after extraction and purification by Quick, Easy, Cheap, Effective, Rugged and Safe (QuEChERS) technique (method 1) and with use of glass chromatographic column packed with alumina (method 2).

Due to the lack of appropriate reference materials representing the composition of tea, the results of PAH determination in black dried tea by HPLC-FLD method proceeded by extraction and purification procedure by QuEChERS technique were compared to the respective results of PAH determination by the method with use of glass chromatographic column packed with alumina [26]. Taking into account the uncertainty estimated for each of the methods, it was found that there were no statistically significant differences between the obtained results (Figure 2); therefore, the QuEChERS method can be recommended for determination of PAHs in tea due to considerably shorter analysis time and less chemical reagent consumption.

**Figure 3:**
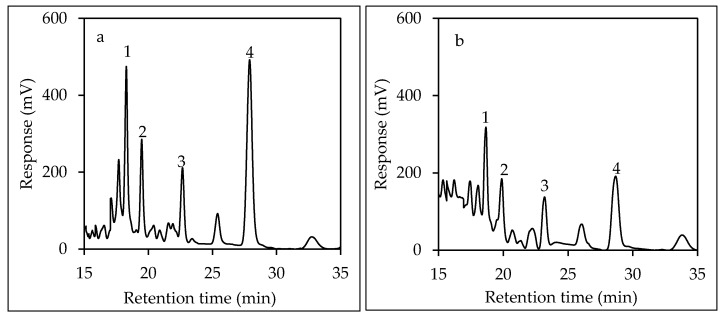
Enlargement of raw HPLC-FLD chromatogram profile of black tea (**a**) and white tea (**b**); benz(a)anthracene (1), chrysene (2), benzo(b)fluoranthene (3), benzo(a)pyrene (4).

Values of the validation parameters (Table 2) allowed for approval to use the method for determining benzo(a)pyrene and sum of the four PAHs in tea. Chromatogram profiles of the determined PAHs in black and white teas are presented in Figure 3a,b, respectively.

The benzo(a)pyrene, benz(a)anthracene, benzo(b)fluoranthene and chrysene concentration values given in Table 3 were corrected for the recoveries obtained for each of the measurement series, in accordance with the requirements of the Commission Regulation (EU) 836/2011 [30]. The recovery values were within the ranges of 51–82%, 66–93%, 55–75% and 64–111% for benzo(a)pyrene, benz(a)anthracene, benzo(b)fluoranthene and chrysene, respectively.

The tea samples contained the four PAHs in the amount exceeding the respective values of LOQ. The lowest amounts of the analysed PAHs (0.76 ± 0.15 μg/kg of BaP and 5.51 ± 1.10 μg/kg of Σ4PAHs) were found in the Rainforest Alliance Certified™ white tea, while the highest ones were found in Assam Indian (209 ± 42 μg/kg of BaP and 756 ± 151 μg/kg of Σ4PAHs) and Yunnan China (158 ± 32 μg/kg of BaP and 770 ± 154 μg/kg of Σ4PAHs) black leaf teas from the same manufacturer/distributor. According to the information on the packaging these products were mixtures of black teas. 

**Figure 4:**
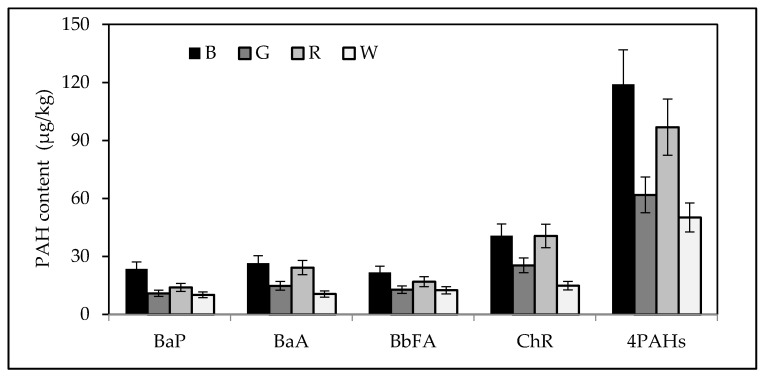
PAH contents (median; μg/kg) of black tea (B), green tea (G), red tea (R) and white tea (W) purchased in the Polish market.

The sum of the four PAHs in black and red teas (119 μg/kg and 97 μg/kg, respectively, expressed as median) were higher than in green and white teas (61.8 μg/kg and 50.1 μg/kg, respectively) (Figure 4). No significant differences were found in benz(a)anthracene, benzo(b)fluoranthene and chrysene contents (expressed as median) between black and red tea samples. The median benzo(a)pyrene content of black tea (23.5 μg/kg) was significantly higher than in green, red and white teas (10.9 μg/kg, 13.9 μg/kg and 10.1 μg/kg, respectively) (Figure 4). For all the tested tea samples, there were large differences between the lowest and the highest PAHs concentrations, in accordance with the results of recent studies (Table 4). The PAH contamination level of teas from the Czech market determined by Drabova et al. [4] was similar to that found in the present study; maximum BaP and Σ4PAHs contents of black tea amounted to 152 μg/kg and 699 μg/kg, respectively. High levels of PAHs in black teas were also reported by Lin and Zhu [7], Iwegbue et al. [31], Lin et al. [32] and Londoño et al. [33] (Table 4). Available literature data indicate that both in EU and in non-EU countries teas with very different levels of polycyclic aromatic hydrocarbons are sold (Table 4). Lin and Zhu [7] analysed tea leaves of each of the processing stages (i.e., before wilting, after wilting, after rolling, after fermentation and after drying) as well as the air above the drying tea leaves in terms of PAH content. The level of the dryer air pollution with polycyclic aromatic hydrocarbons was about one hundred times higher than this of the outdoor air [7]. It was found that large amounts of PAHs in the air resulted from its heating by the burning of pine wood. The PAHs were then consequently absorbed by the tea leaves. The median values of BaA for black and red tea did not differ significantly. There were also no significant differences in median BaFA, Chr and Σ4PAHs contents between black and red tea samples (Figure 4). The level of contamination of tea with PAH compounds reported by Londoño et al. [33] for red tea sold in Argentina, as well as the BaP content of red tea from the Polish market reported by Ciemniak and Mocek [34] do not differ significantly from the results of the present study. On the other hand, the BaP, BbFA and Chr contents determined by Sadowska-Rociek et al. [21] in different types of tea (Table 4) were significantly lower than these of our study.

The level of contamination of both green and white tea with PAHs was significantly lower than that of black tea; this is in accordance with the reports of other researchers (Table 4). The determined BaP content of green tea (4.3–24.8 μg/kg) is similar to that of Drabova et al. [4], Ziegenhals et al. [10] and Ciemniak and Mocek [34] (Table 4). On the other hand, markedly higher amounts of PAHs were determined by Londoño et al. [33] in tea samples from Argentina. The high levels of contamination of some white teas with PAHs may be due to their different processing as compared to the other types of tea. Tea buds are harvested in early spring, when high PAHs concentrations are possible due to emission sources of the heating season. Moreover, at the beginning of the growing season the young tea leaves contain the highest amount of the lipophilic essential oils which facilitate the absorption of PAHs [35].

### 3.2. PAH Content of Tea Infusion

Prior to determining the BaP, BaA, BbFA and Chr content in tea infusions, the validation parameters of the method were tested. For each of the studied compounds, the limit of detection (LOD), the limit of quantification (LOQ), the recovery and the repeatability were determined (Table 5).

Due to low levels of the PAHs expected in tea infusions, the enrichment of the latter with a mixture of BaP, BaA, BbFA and Chr was performed at the lowest possible concentration level. It was found that it is possible to determine the four PAHs in tea infusions containing no more than 0.15 ng/mL, 0.15 ng/mL, 0.10 ng/mL and 0.15 ng/mL or BaP, BaA, BbFA and Chr, respectively (Table 5).

**Figure 5:**
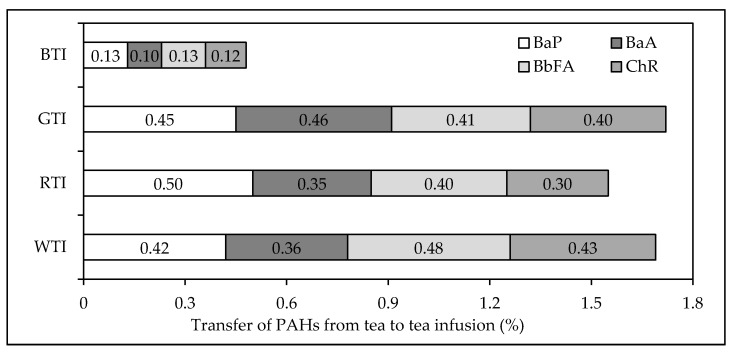
Percentage transfer of PAHs from tea to tea infusion: BTI—black tea infusion, GTI—green tea infusion, RTI—red tea infusion, WTI—white tea infusion.

Determination of PAHs in 28 tea infusions was performed according to the procedure presented in Figure 1. PAH contents above LOQ were found only in two black tea infusion samples, two green tea infusion samples, one red tea infusion sample and three white tea infusion samples. For these samples the transfer of the four PAHs from tea to tea infusion was calculated. The percentage transfer of sum of the four PAHs from tea to tea infusion for red, white and green tea samples were found to be similar to each other (1.55%, 1.69% and 1.72%, respectively), while for black tea samples it was significantly lower (0.48%) (Figure 5). Duedahl-Olesen et al. [36] have found the presence of the four PAHs in 55% of the analysed black tea samples. They reported that the maximum level of transfer of sum of the four PAHs amounted to 2.3%, with its mean value of 0.86%. Lin et al. [6] reported, in turn, that the percentage transfer of BaP from China black tea of 246 μg BaP/kg to tea infusion was 0.39%. Lin et al. [6] found that tea variety, tea/water ratio and brewing time affect transfer of 12 polycyclic aromatic hydrocarbons from tea to tea infusion. Moreover, they reported that washing tea immediately before brewing as well as brewing the tea within uncovered cup reduced the percentage transfer of the PAHs up to 30%. It was also found that significantly more polycyclic aromatic hydrocarbons of from two to four aromatic rings are transferred from tea to tea infusion than these of five or six aromatic rings. This phenomenon can result from lower water solubility of PAHs with higher molecular weights. Viñas et al. [37] reported the presence of PAH compounds with two or three aromatic rings in white, green and red tea infusions, while benzo(a)pyrene, benz(a)anthracene, benzo(b)fluoranthene and chrysene were not found. Similarly, BaA and Chr were not detected in tea infusions prepared from black, green and red tea available on the Spain market [38]. Schulz et al. [39] detected BaP and BbFA in the amounts of 0.009 μg/L and 0.006 μg/L, respectively, only in one tea infusion prepared from black tea purchased on the German market. The BaP contents of tea infusions reported by Ciemniak and Mocek [34] were from 0.4 ng/L to 18.7 ng/L for 2.9–63.1 μg of BaP per one kilogram of dried black, green, red and white teas available on the Polish retail market. Maximum BaA, BbFA and Chr contents in these tea infusions were 32.7 ng/L, 17.6 ng/L and 36.4 ng/L, respectively.

**Figure 6:**
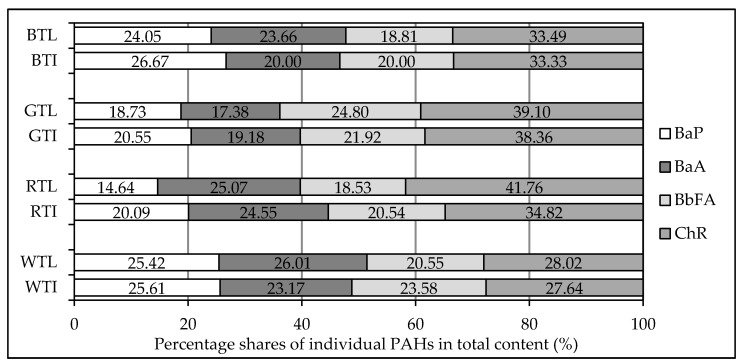
Percentage shares of individual PAHs in total content: BTL—black tea leaves, BTI—black tea infusion, GTL—green tea leaves, GTI—green tea infusion, RTL—red tea leaves, RTI—red tea infusion, WTL—white tea leaves, WTI—white tea infusion.

Figure 6 shows the percentage share of individual PAHs in their sum, both for tea leaves and tea infusions. The presented data show that the dominant compound in both tea leaves and tea infusions is chrysene. Its share ranges from 28%, in the case of white tea leaves, to almost 42% in red tea leaves. In the case of tea infusions, the lowest share of chrysene was found for white tea, while the highest one was found for green tea. Noteworthy also is the percentage share of benzo(a)pyrene, which had been a marker of the occurrence of PAH in food for many years. In the case of both black and white tea leaves and infusions, BaP’s share amounted to about 25%; however, in other tea varieties, it was about 20% and below. The lowest share of BaP was found for red tea leaves and infusion (Figure 6).

## 4. Conclusions

The QuEChERS technique used for the extraction and purification of tea samples for the determination of benzo(a)pyrene, benz(a)anthracene, benzo(b)fluoranthene and chrysene may be successfully used after optimization and validation of the method. Teas commercially available on the Polish market are characterized by great diversity in terms of contamination with polycyclic aromatic hydrocarbons. The highest concentrations of BaP and Σ4PAHs—209.4 ± 41.9 μg/kg and 755.5 ± 151.1 μg/kg, respectively—were found for black leaf tea. The transfer of sum of the four PAHs from tea to tea infusion was 0.48% and 1.55–1.72% for black tea and for red, white and green teas, respectively.

## Figures and Tables

**Table 1:** PAH contents of black and white tea and tea infusions depending on various tea to water ratios (evaluated to optimize the method of determination of PAHs in tea infusion).

Kind of PAH	PAH Content of Tea (μg/kg)	PAH Content of Tea Infusion (ng/mL)			
		Tea Weight (g)/Water Volume (mL)			
		2/250	20/250	10/100	10/50
**black tea**					
BaP	209	<LOQ	<LOQ	0.16	0.25
BaA	174	<LOQ	<LOQ	0.11	0.18
BbFA	142	<LOQ	<LOQ	0.13	0.19
Chr	231	<LOQ	0.18	0.20	0.33
**white tea**					
BaP	23.9	<LOQ	<LOQ	<LOQ	0.15
BaA	38.4	<LOQ	<LOQ	<LOQ	0.15
BbFA	25.6	<LOQ	<LOQ	<LOQ	0.11
Chr	42.7	<LOQ	<LOQ	<LOQ	0.16

LOQ—limit of quantification; BaP—benzo(a)pyrene; BaA—benz(a)anthracene; BbFA—benzo(b)fluoranthene; Chr—chrysene.

**Table 2:** Validation parameters of the method of determination of PAHs in tea.

Parameter		Kind of PAH			
		BaP	BaA	BbFA	Chr
Linearity—correlation coefficient		0.9999	1.0000	0.9999	1.0000
Sensitivity (slope) by regression equation		2.451	9.050	7.398	3.667
Limit of detection (LOD) (μg/kg) (n = 10)		0.25	0.15	0.15	0.25
Limit of quantification (LOQ) (μg/kg) (n = 10)		0.75	0.50	0.50	0.75
Recovery (%)	Level I (5.00 μg/kg) (n = 6)	82.25	93.30	75.00	71.35
	Level II (40.00 μg/kg) (n = 6)	50.75	66.70	57.05	64.70
	Level III (250 μg/kg) (n = 6)	67.20	76.20	69.90	75.80
Repeatability RSD_r_ (%) (n = 20)		8.8	5.5	2.2	3.6
HORRAT_r_		0.61	0.34	0.15	0.25

Explanations: n—number of determinations; Repeatability RSD_r_—repeatability relative standard deviation; HORRAT_r_—the observed RSDr divided by the RSDr value estimated from the modified Horwitz equation [30]; BaP—benzo(a)pyrene; BaA—benz(a)anthracene; BbFA—benzo(b)fluoranthene; Chr—chrysene.

**Table 3:** Ranges and means of PAH contents (μg/kg) in the selected teas purchased in the Polish market.

Tea Type	Kind of PAH				
	BaP	BaA	BbFA	Chr	Σ4PAHs
Black tea (n = 10)	3.96–209.36	7.58–187.00	4.51–145.10	14.80–280.00	33.12–770.10
	51.13	51.53	41.08	75.06	218.80
Green tea (n = 6)	4.30–24.82	5.62–23.03	7.27–46.53	9.80–61.20	29.62–153.27
	13.34	15.10	18.25	28.91	75.96
Red tea (n = 6)	7.00–18.09	15.81–30.99	9.13–22.89	23.80–51.61	55.74–120.41
	12.93	23.24	16.25	37.79	90.21
White tea (n = 6)	0.76–26.55	1.01–38.41	0.85–25.61	2.89–42.73	5.51–130.74
	11.52	14.87	13.11	18.34	57.40

Explanations: Minimum–maximum and mean values are presented; Σ4PAHs—sum of benzo(a)pyrene, benz(a)anthracene, benzo(b)fluoranthene and chrysene; n—number of samples.

**Table 4:** Comparison of results generated within presented study with similar studies.

Tea Type	Sampling Market	Number of Samples		Kind of PAH				Reference
			BaP	BaA	BbFA	Chr	Σ4PAHs	
Black tea	Poland	10	3.9–209	7.6–187	4.5–145	14.8–280	33–770	present study
	Poland	7	n.d.	2.4–47	1.9–8.1	1.6–18	-	[21]
	Poland	9	2.9–63	-	-	-	-	[34]
	Czech Republic	18	0.2–152	1.4–196	0.9–123	3.9–229	7.4–699	[4]
	Argentina	27	0.2–93	0.2–63	0.1–68	2.5–109	4.1–332	[33]
	Denmark	10	0.30–32	-	-	-	2.8–115	[36]
	China	2	20.1–246	-	-	-	-	[6]
	Nigeria	4	n.d.–137	n.d.–44	n.d.–27	n.d.–55	-	[31]
	Germany	11	0.8–14	1.3–13	1.5–8.1	3.4–18	9.0–44	[10]
Green tea	Poland	6	4.3–25	5.6–23	7.3–47	9.8–61	29–153	present study
	Poland	7	n.d	11–19	1.8–2.0	2.8–3.7	-	[21]
	Poland	3	5.6–31	-	-	-	-	[34]
	Czech Republic	18	0.2–18	0.7–28	0.7–24	2.9–42	4.5–102	[4]
	Argentina	14	0.4–61	0.7–74	0.15–67	4.6–154	8.0–356	[33]
	China	1	6.8	-	-	-	-	[6]
	Nigeria	3	n.d.	n.d.	n.d.–27	n.d.–55	-	[31]
	Germany	11	1.6–33	1.8–40	2.2–33	6.7–62	12–168	[10]
Red tea	Poland	6	7.0–18	16–31	9.1–23	24–52	55–120	present study
	Poland	3	n.d	n.d.–33	n.d.	3.2–12	-	[21]
	Poland	3	9.7–15	-	-	-	-	[34]
	Argentina	7	0.7–16	0.5–41	0.5–25	5.8–64	7.4–127	[33]
White tea	Poland	6	0.8–27	1.0–38	0.8–26	2.9–42	5.5–131	present study
	Poland	5	n.d.	2.4–17	2.2	12–19	-	[21]
	Poland	3	7.6–49	-	-	-	-	[34]
	Argentina	1	22	16	19	35	92	[33]
	Germany	3	11	14–80	14–45	19–95	59–80	[10]

Explanations: Minimum–maximum and mean values (μg/kg) are presented; Σ4PAHs—sum of benzo(a)pyrene, benz(a)anthracene, benzo(b)fluoranthene and chrysene; “-”—no data; n.d.—not detected.

**Table 5:** Validation parameters of the method of determination of PAHs in tea infusion.

Parameter	Kind of PAH			
	BaP	BaA	BbFA	Chr
Linearity—correlation coefficient	0.9999	0.9999	0.9999	0.9999
Limit of detection (LOD) (ng/mL) (n = 10)	0.05	0.03	0.03	0.05
Limit of quantification (LOQ) (ng/mL) (n = 10)	0.15	0.10	0.10	0.15
Recovery Level I (0.18 ng/mL) (n = 6) (%)	110.56	105.00	107.22	99.44
Repeatability RSD_r_ (%) (n = 10)	8.0	4.4	5.1	6.5
HORRAT_r_	0.55	0.30	0.35	0.45

Explanations: n—number of determinations; Repeatability RSD_r_—repeatability relative standard deviation; HORRAT_r_ = the observed RSDr divided by the RSDr value estimated from the modified Horwitz equation [30].
